# Rates of ventilator-associated pneumonia in critical care units in three Arabian Gulf countries; six-year surveillance study

**DOI:** 10.1186/2047-2994-4-S1-P245

**Published:** 2015-06-16

**Authors:** A El-Saed, HH Balkhy, H Alansari, A Althaqafi, J Alsalman, Z Al Maskari, A El Gammal, W Al Nasser, A Al Jardani, SS Al-Abri

**Affiliations:** 1 Infection Control, King Abdulaziz Medical City, Riyadh, Saudi Arabia; 2 Infection Control, Salmaniya Medical Complex, Manama, Bahrain; 3 Infection Control, King Abdulaziz Medical City, Jeddah, Saudi Arabia; 4 Infection Control, Royal Hospital, Muscat, Oman; 5 Infection Control, King Abdulaziz Hospital, Al Hassa, Saudi Arabia; 6 Infection Control, Imam Abdulrahman bin Faisal Hospital, Dammam, Saudi Arabia

## Introduction

Data estimating the rates of ventilator-associated pneumonia (VAP) in critical patients in Gulf Cooperation Council (GCC) countries are either limited in some countries or completely lacking in other countries.

## Objectives

To estimate VAP rates in GCC hospitals and to compare such rates with published reports of US National Healthcare Safety Network (NHSN) and International Nosocomial Infection Control Consortium (INICC).

## Methods

VAP rates and ventilator utilization between 2008 and 2013 were calculated from aggregated VAP surveillance data using NHSN methodology pooled from 6 hospitals in three GCC countries; Saudi Arabia, Oman, and Bahrain. Standardized infection ratio (SIR) of VAP in GCC hospitals were compared with published reports of NHSN and INICC.

## Results

A total 368 VAP events were diagnosed during 6 years of surveillance covering 76,749 ventilator days and 134,994 patient-days. The overall VAP rate was 4.8 per 1000 ventilator days (95% CI ranged between 4.3 and 5.3) with an overall ventilator utilization of 0.57. The VAP rates showed a wide variability between different types of intensive care units (ICUs) and were decreasing overtime. After adjusting for the differences in ICUs types in both sides, the risk of VAP in GCC hospitals was 217% higher than NHSN hospitals but 69% lower than INICC hospitals. Similarly, after adjusting for the differences in weight groups in neonatal ICUs in both sides, the risk of VAP in GCC hospitals was 69% higher than NHSN hospitals but 78% lower than INICC hospitals. Approximately 55% of the VAP events were diagnosed based on clinical criteria, which was very similar to published frequencies from similar ICUs in NHSN hospitals.

## Conclusion

Current findings is considered the first regional VAP benchmark. The risk of VAP in adult and neonatal ICU patients in GCC countries is probably higher than the US but lower than many developing countries.

## Disclosure of interest

None declared.

